# Pervasive Monitoring—An Intelligent Sensor Pod Approach for Standardised Measurement Infrastructures

**DOI:** 10.3390/s101211440

**Published:** 2010-12-13

**Authors:** Bernd Resch, Manfred Mittlboeck, Michael Lippautz

**Affiliations:** 1 Research Studios Austria, Schillerstrasse 30, 5020 Salzburg, Austria; E-Mails: manfred.mittlboeck@researchstudio.at (M.M.); michael.lippautz@researchstudio.at (M.L.); 2 Research Affiliate, MIT SENSEable City Lab, 77 Massachusetts Avenue, Cambridge, MA 02139, USA

**Keywords:** pervasive sensing, standardised geo-sensor web, intelligent sensor pod, Complex Event Processing

## Abstract

Geo-sensor networks have traditionally been built up in closed monolithic systems, thus limiting trans-domain usage of real-time measurements. This paper presents the technical infrastructure of a standardised embedded sensing device, which has been developed in the course of the Live Geography approach. The sensor pod implements data provision standards of the Sensor Web Enablement initiative, including an event-based alerting mechanism and location-aware Complex Event Processing functionality for detection of threshold transgression and quality assurance. The goal of this research is that the resultant highly flexible sensing architecture will bring sensor network applications one step further towards the realisation of the vision of a “digital skin for planet earth”. The developed infrastructure can potentially have far-reaching impacts on sensor-based monitoring systems through the deployment of ubiquitous and fine-grained sensor networks. This in turn allows for the straight-forward use of live sensor data in existing spatial decision support systems to enable better-informed decision-making.

## 1. Introduction

“In the next century, planet Earth will don an electronic skin. It will use the Internet as a scaffold to support and transmit its sensations. This skin is already being stitched together. It consists of millions of embedded electronic measuring devices. … These will probe and monitor cities and endangered species, the atmosphere, … fleets of trucks, our conversations, our bodies—even our dreams [1].”

Following this comprehensive vision formulated by Neil Gross in 1999, it can be assumed that sensor web deployments (see Subsection 3.2 for a disambiguation of the term “sensor web” *vs*. “sensor network”) will increase dramatically within the coming years, as pervasive sensing has recently become feasible and affordable. This enriches knowledge about our environment with previously uncharted real-time information layers.

Of specific interest in this paper is the concern that most sensor networks are being built up in monolithic and specialised application-centred measurement systems. Subsequently, there is a clear gap between sensor network research and largely heterogeneous end user requirements. Sensor network research is usually dedicated to a long-term vision, which tells a compelling story about potential applications. On the contrary, the actual implementation is often not more than a very specific use case oriented closed system without taking into account technical and conceptual issues such as interoperability, sustainable development, portability, or combination with established data analysis systems. Therefore, the availability of geo-sensor networks is growing but still limited [2].

Generally speaking, a key requirement for achieving meaningful analysis results in sensor-based systems is high quality of underlying data in terms of *accuracy*, *completeness* and *topicality*. While the first two parameters have traditionally received a lot of attention, the topicality parameter is still neglected in most cases. This is partly due to the fact that geo-data is per definition historic and so far, geospatial processing has focused on analysing static data, with low temporal fluctuations [3].

Furthermore, all components of measurement systems are currently undergoing great performance enhancement combined with drastic price reduction [4]. This will particularly be so if monitoring policies enforced by super-national directives move from a mathematical modelling base to a more pervasive monitoring structure. Thus, it can be assumed that sensor web deployments will increase dramatically within the coming decade.

This raises the issue that real-time integration of heterogeneous data sources will be of particular importance as a broad spectrum of diverse actors establish their systems, often measuring the same parameters. This paper presents the sensor web part of the Live Geography approach [3], which seeks to tackle the challenges of heterogeneous data sources and manifold analysis processes with an open sensing infrastructure for monitoring applications. The overall infrastructure makes extensive use of open (geospatial) standards throughout the entire process chain—from sensor data integration to analysis, Complex Event Processing (CEP), alerting, and finally visualisation.

This paper is structured as follows: after a short presentation of related work (Section 2), a brief status quo is given on pervasive sensing and sensor web technology (Section 3). This is followed by a detailed description of the Live Geography concept for standardised embedded sensing (Section 4) and its implementation of an intelligent sensor pod (Section 5). The final sections of the paper deal with a discussion of standardisation needs and processes (Section 6), and a short conclusion and future outlook (Section 7).

## 2. Related Work

There are a number of approaches establishing pervasive sensor web applications. In [5] a sensing infrastructure that attempts to combine sensor systems and GIS-based visualisation technologies is presented. The sensing devices, which measure rock temperature at ten minute intervals, were developed focusing on optimised resource usage, including data aggregation, power consumption, and communication within the sensor network. In its current implementation, the infrastructure does not account for geospatial standards in sensor observations. The visualisation component uses a number of open standards (Open Geospatial Consortium (OGC) Web Map Service (WMS) and Web Feature Service (WFS)) and open-source services (UMN Map Server, Mapbender).

Another sensing infrastructure is described in [6]. The CitySense project uses an urban sensor network to measure environmental parameters and is thus the data source for further data analysis. The project focuses on the development of a city-wide sensing system using an optimised network infrastructure. An important parallel with the work presented in this paper is that CitySense also considers the requirements of sensor network setup in restrictive environments.

King’s College London established an urban sensor network for air quality monitoring. The London Air Quality Network (LAQN) [7] was formed in 1993 to coordinate and improve air pollution monitoring in London. Increasingly, measurement data are being supplemented by measurements from local authorities surrounding London, thereby providing an overall perspective of air pollution in South East England. Currently, the LAQN consists of about 150 monitoring sites being a very promising approach to ubiquitous sensing as it also offers on-the-fly creation of statistic graphs, time series diagrams and wind plots.

The SenseWeb project aims to establish a Wikipedia-like sensor platform [8]. The project seeks to allow users to include their own sensors in the system and thus leverage the “community effect”, building a dense network of sensors by aggregating existing and newly deployed sensors within the SenseWeb application. Although the authors discuss data transformation issues, data fusion, and simple geospatial analysis, the system architecture is not based on open (geospatial) standards, only standard web services. The web portal implementation, called SensorMap, uses the Sensor Description Markup Language (SDML), an application-specific OGC SensorML dialect.

Summarising, these network developments do not make use of open standards as a whole, meaning that they are built up in proprietary systems, although sensor data are accessible over the internet. Despite this shortcoming, the described sensor networks can be considered stand-alone solutions of great local significance, though limiting trans-regional inter-linkage with other similar approaches.

## 3. Pervasive Sensing—Status Quo

Pervasive sensing and ubiquitous monitoring are critical processes to ensure public safety including the state of the national infrastructure, to set up continuous information services, and to provide input for spatial decision support systems [3].

### 3.1. Closed Monolithic Systems Limit Trans-Domain Sensing Capacities

However, establishing an overarching monitoring infrastructure is not trivial. Currently, different authorities with heterogeneous interests each implement their own monolithic systems to achieve very specific goals in areas like air quality monitoring, flood water prediction or traffic management.

**Figure 1.**
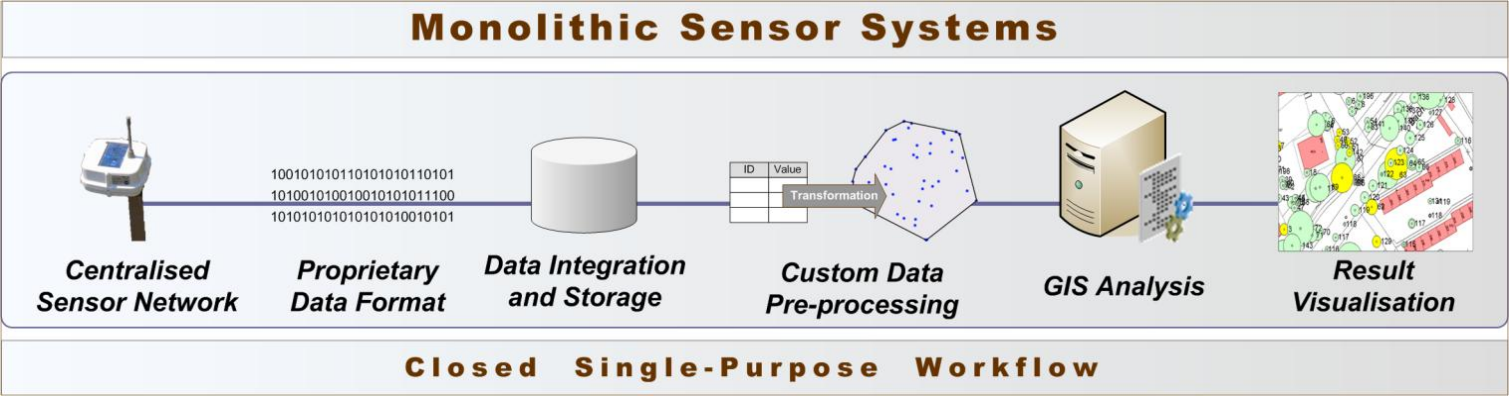
Current Approach—Monolithic Single-purpose Sensor Systems.

These measurement systems are mostly built up in a minimalistic—though often short-term cost-optimised—manner resulting in a hard-wired workflow, as shown in Figure 1. Such systems mostly serve a single purpose and can often not be adapted to other end applications. This also means that the components within the overall process are chained together in a fixed way (“black box” or “stove-pipe”), which prevents re-use of single modules.

These single-purpose measurement systems are typically characterised by a centralised sensor network sending data in a proprietary format, which prevents distributed architectures and interoperability with other systems. The gained measurements are stored in a database, which can often become a bottle-neck in the overall workflow when dealing with large amounts of data.

The fact that these systems tend to be deployed in an isolated and uncoordinated way means that the automatic assembly and analysis of these diverse data streams is impossible. However, making use of all available data sources is a prerequisite for holistic and successful monitoring for broad decision support. This applies to emergency situations as well as to continuous observation of environmental parameters. Thus, we developed the Live Geography approach, which tries to standardise the whole process chain shown in Figure 1 [3].

### 3.2. Sensor Web Technology

Over the last few years, sensor webs have been a rapidly emerging technology, which serves for gathering measured data and combining them to generate an overall combined result. For the formation of such a sweeping vision of a “digital skin for planet Earth” as envisaged in [1], and described in the introductory section, sensors have to be inter-linked and easily accessible to provide live sensor data. This enables near real-time decision support in diverse areas such as environmental monitoring, medical emergencies, disaster management or public safety.

Generally speaking, sensor webs are a network of intra-communicating sensors, which might be of a different type, used to monitor the environment. Monitored parameters are manifold, including temperature, precipitation, different substances in the atmosphere, processes within the human body, industrial control functions, and innumerable more.

In contrast to ordinary sensor networks, sensor webs are characterised by three main criteria. The first characteristic is **interoperability**, which means that different types of sensors should be able to communicate with each other and produce a common output (see OGC Sensor Web Enablement (SWE) description in Subsection 4.2). The requirement of **scalability** implies that new sensors can be easily added to an existing topology without necessitating aggravating changes in the present hardware and software infrastructure. Finally, **intelligence** means that the sensors are able to think autonomously to a certain degree, which could e.g., result in a data processing ability in order to only send a filtered sub-set of the data as required by the user.

Within sensor webs, information is accessed via attribute-based naming instead of explicit addressing, which enables resource-sparing and effective data retrieval. Depending on the capabilities of the sensor, also a history of measured values might be saved resulting in the aptitude to create modular outputs for a certain period of time. This also enables the generation of time series of sensor values originating from a combination of single measurements, which is important for generating motion vectors or mean values over a particular interval to perform plausibility checks.

## 4. “Live Geography” Embedded Standardised Geo-sensor Web Concept

Geo-sensor webs are the essential data source for monitoring applications and real-time information services. Section 2 illustrated a number of recent sensor network deployments, showing that research areas are highly heterogeneous; as well in sensor web research itself as in their application fields.

### 4.1. Live Geography Specific Sensor Web Requirements

The design of a sensor network is defined by a number of parameters such as the operating environment, miniaturisation requirements, energy consumption, bandwidth utilisation or connectivity. These factors are highly application-specific and can thus not be uniquely specified for a general architecture like the Live Geography infrastructure.

However, a fundamental prerequisite for pervasive sensor webs is interoperability, which concerns data structures, measurement transmission, sensor queries and alerting functionality. Thus, the OGC Sensor Web Enablement (SWE) initiative has been chosen to assure interoperability throughout the Live Geography architecture. This choice was taken because of SWE’s comprehensiveness (it serves the whole process chain), its broad development support as well from academia as from industry, its rapid advancement and introduction as official standards, and its maturity, which it has mainly gained through the last two years.

The most essential SWE standards for monitoring applications are the Sensor Observation Service (SOS) for sensor data provision, Observations and Measurements (O&M) for encapsulating measurements into a standardised XML-based format, and the Sensor Model Language (SensorML) for describing sensor platforms. Furthermore, the Sensor Alert Service (SAS) plays an important role for sending event-triggered alerts, e.g., in case of threshold transgression.

The actual implementation of the geo-sensor web is greatly application-specific according to the unique requirements of every single deployment. Thus, the Live Geography approach only suggests broad interoperability, which is achieved by the extensive use of the OGC SWE standards, whereas other sensor network challenges have to be solved as the case arises.

A central design aspect in the Live Geography approach was the support for both stationary and mobile sensors. Stationary sensors are deployed at a fixed position where their geographical location is only measured once. The position can be—but does not have to be—transmitted along with every broadcasted data set in the same manner as with mobile sensors. In contrast to stationary sensors, mobile ones have to record their current position periodically in order to make measurement results meaningful. The main challenges in the development of a sensor pod supporting stationary and mobile deployments are optimised data transmission capabilities, communication availability, resource-saving operation modes and optimised database design (finding the best possible trade-off between simplicity and normalisation). The implementation described in Section 5 addresses these issues.

### 4.2. Sensor Web Enablement Initiative

SWE aims to make different types of sensors discoverable, accessible and possibly controllable over the web, and thus to enable the creation of interoperable and scalable sensor webs, which are built up in a service-oriented fashion [9]. SWE comprises seven OGC standards and interoperability programme reports, SensorML, O&M, SOS, SAS, the Transducer Model Language (TransducerML), the Sensor Planning Service (SPS) and the Web Notification Service (WNS). The following subsections describe the four standards/specifications, which the intelligent sensing device presented in this paper implements (SOS, SAS, O&M and SensorML).

#### 4.2.1. Sensor Model Language

The Sensor Model Language (SensorML) is a general schema for describing functional models of the sensor. It provides an Extensible Markup Language (XML) schema for defining the geometric, dynamic and observational properties of a sensor. Thus, SensorML serves for discovering different types of sensors, supporting processing and analysis of the retrieved data as well as the geo-location of observed values [10]. SensorML encodes all processes and components as application schema of the Feature model in the Geography Markup Language (GML) Version 3.1.1. An important capability of SensorML that has to be mentioned considering the background of this research is the formation of sensor classes, *i.e.*, sensing devices with the same properties. In SensorML, a sensor array defines a set of devices of the same type at different locations, whereby a sensor group describes several sensors that operate together to provide one collective observation.

#### 4.2.2. Observations and Measurements

Observations and Measurements (O&M) provides a description of sensor observations and measurements in the form of general models and XML encodings. The term observation is defined as “an action with a result which has a value describing some phenomenon”. The O&M standard labels several terms for the measurements themselves as well as for the relationship between them, whereby the extent is limited to measurement results, which are expressed as quantities, categories, temporal or geometrical values as well as arrays or composites of these [11].

It shall be stated that despite OGC’s tendency towards using the geographical position as the central and connecting element in geospatial standards, the location parameter is considered a regular measurement within O&M. This means that the position is equipollent with other measurands such as time, air temperature or satellite images.

#### 4.2.3. Sensor Observation Service

**Figure 2.**
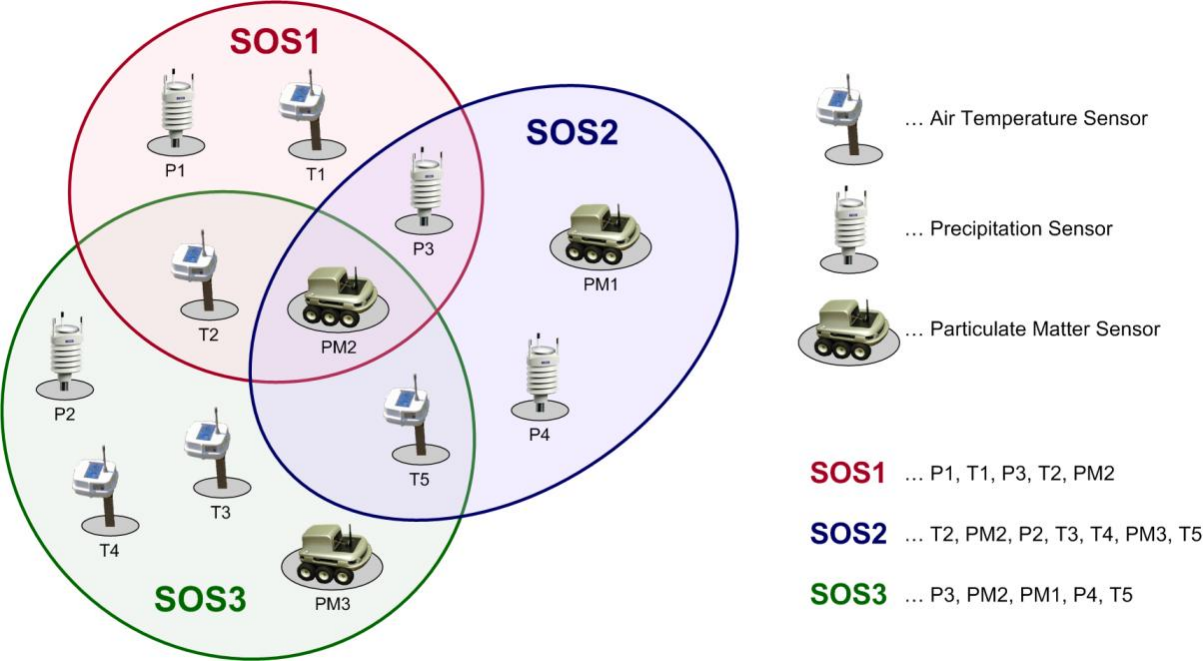
General SOS Usage Concept (Adapted from [12]).

The Sensor Observation Service (SOS) provides a service interface for standardised access to sensor data and metadata. In other words, the SOS groups a collection of possibly heterogeneous sensors, as illustrated in Figure 2, and provides their measurements via a standardised service interface.

The SOS specification defines the operations offered by a specific sensor, whereat the minimum collection of methods comprises *GetCapabilites*, *DescribeSensor* and *GetObservation*, which return information about the observations and measurements supported by the SOS. SOS references the O&M specification for encoding sensor observations, and the SensorML and TransducerML specifications for modelling sensors and sensor systems [12].

#### 4.2.4. Sensor Alert Service

The OGC Sensor Alert Service (SAS) specifies interfaces (not a service in the traditional sense) enabling sensors to advertise and publish alerts including according metadata. While SOS allows for pull-based data queries, SAS provides data in a push-based manner. Thus, SAS can be used by clients to subscribe for sensor data alerts with some spatial and property-based constraints. Also, sensors can be advertised to the SAS allowing clients to subscribe for the sensor data via the SAS, as shown in Figure 2. SAS, which is currently in its version 0.9.0, is not released as an official OGC standard [13].

**Figure 3.**
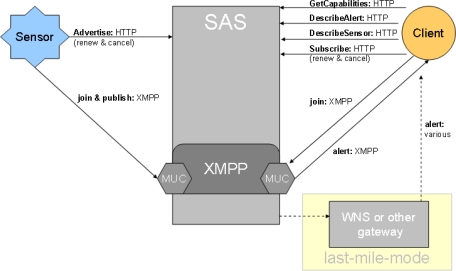
General SAS Functionality [13].

As Figure 3 illustrates, SAS uses the Extensible Messaging and Presence Protocol (XMPP) for the delivery of sensor notifications. Thus, SAS leverages an XMPP server, which can be embedded directly in the SAS or act as a separate service. SAS notifications are provided via a Multi User Chat (MUC) for each registered sensor. To receive notifications, a client has to join the specific MUC.

#### 4.2.5. Sensor Alert Service *vs*. Sensor Event Service

The OGC Sensor Event Service (SES) constitutes another specification (to date an OGC Discussion Paper) for event-driven data transmission. In contrast to SAS, SES specifies a service interface for filtering sensor data according to pre-defined filter criteria, whereby the filtering process itself operates on (sensor) data or on events. In this context, events are defined as “actions that occur at an instant or over an interval of time”. SES defines three levels of filtering:

- syntactic filtering of sensor data
- filtering on single data sets (sensor measurements)
- filtering of data streams (“data fusion”)

Generally speaking, the provided filtering capabilities of SAS and SES are clearly on a different scale. While SAS only provides predefined filter capabilities, SES allows for the dynamic creation of custom filter criteria. On the one hand static criteria and definitions may restrict the freedom of an application and its use-cases, on the other hand they are much easier to implement and can therefore be used on light-weight systems such as embedded devices. The possibility of defining dynamic criteria in SES provides more flexibility and can be adopted for a broader range of high-level applications. It shall be noted that this kind of flexibility comes with the price of more complex implementations. Taking these facts into consideration, SES may provide more flexibility using dynamic criteria, but in its current specification, it is hardly feasible to implement it in small-scale systems.

Another difference *versus* SAS is that SES does not define a transport protocol for performing filtering operations, whereas SAS specifies dedicated protocol usage; HTTP is used for advertising sensor data and client requests, whereas the Extensible Messaging and Presence Protocol (XMPP) is used for push based alerting. More detail about the SES standard can be found in [14].

Generally, SES (currently in version 0.0.1) is designed as a functional extension of SAS (currently in version 0.9) in terms of event (stream) processing. In addition to SAS, SES not only specifies the interface for advertising and publishing alerts, but also for filtering operations. Consequently, for providing the same functionality like the SES, SAS has to be coupled with an event processing engine, which creates events. Again, after this processing, results can be published via the SAS interface.

#### 4.2.6. SWE Common

An important ongoing effort in SWE development is the establishment of a central SWE Common specification. Its goal is to optimise common usage and maximise reusability by grouping common elements for several standards under one central specification. Thus, it aims to reduce redundancy between the seven standards. In other words, the SWE Common namespace specification aims at collecting elements, which are used in more than one standard of the SWE family. SWE Common will mainly comprise very general elements such as counts, quantities, time elements or simple generic data representations. SWE Common is currently part of the SensorML standard. However, it is foreseen to make SWE Common a separate stand-alone document as an integral component of the SWE initiative.

### 4.3. Location-Aware Event-Based Alerting

Apart from standardised data transmission and provision, a special focus in the Live Geography approach is the extension of Complex Event Processing (CEP) functionality by spatial components. In a geographic context, CEP can e.g., serve for detecting threshold exceedances, for geo-fencing implementations, for investigating spatial clusters or as well for assuring data quality. To guarantee real-time measurement quality (e.g., to detect sensor failures or measurement outliers), location-enabled CEP technology can be used following Waldo Tobler’s first law of geography stating that near features are more related then distant features.

**Figure 4.**
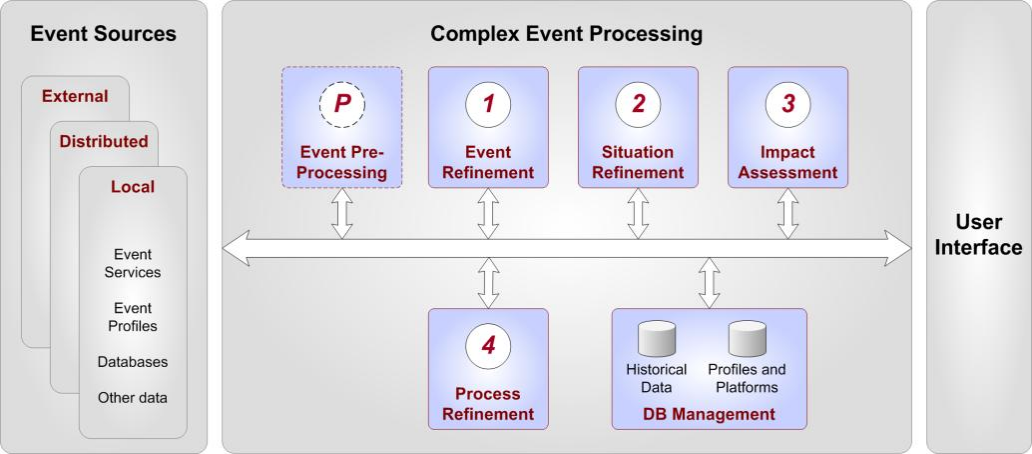
General CEP Architecture with Data Processing Levels (Adapted from [15]).

Generally speaking, CEP is a technology that extracts knowledge from distributed systems and transforms it into contextual knowledge. Since the information content of this contextual knowledge is of higher quality than in the original raw data, business decisions that are derived from it can be more accurate. A general CEP architecture is shown in Figure 4, which has been adapted from [15].

The CEP processing chain is divided into five levels that represent processing steps and the database management component, which is responsible for handling data and profiles. Since individual systems may have different requirements, the implementation depth will vary from system to system. The following list notes the basic requirements for each level, according to [15].

- Level P: Pre-processing—Before the actual processing, the input data is normalised, validated and eventually pre-filtered. Although this is an essential step, it is technically not part of the CEP process and therefore marked as “Level P”.
- Level 1: Event Refinement—This component’s task is to track and trace an event in the system. An event’s characteristics, such as data, behaviour and relationships, are translated into event attributes, *i.e.*, prepared for further processing.
- Level 2: Situation Refinement—The central part in the CEP workflow is the Situation Refinement, where the actual processing of simple and complex events takes place. This analysis includes mathematical algorithms, which comprise not only computing boundaries for certain values, but also matching them against patterns and historical data.
- Level 3: Impact Assessment—This stage deals with the simulation of outcomes. It takes various scenarios and simulates them by taking cost factors and resources into account.
- Level 4: Process Refinement—Finally, the last level deals with the interaction between the CEP system and business processes. It defines a feedback loop to control and refine business processes. While this could include integration and automatic controlling of processes, e.g., within a geo-workflow, it may also just be the creation of alerting messages or business reports.

CEP has originated and traditionally been implemented in the financial and economic sectors to predict market developments and exchange rate trends. In these areas, CEP patterns emerge from relationships between the factors time, cause (dependency between events) and aggregation (significance of an event’s activity towards other events). In a location-aware CEP these aspects are extended by additional parameters indicating spatial information of the event.

The new approach of integrating the “location” parameter into CEP systems, which is pursued in this paper, allows for rule-based data processing of measurement data and for creating complex events accordingly. These can be related to time (temporal validity), space (e.g., geo-fencing with geographic “intersect”, “overlap” or “join” operations) or measurement parameters (e.g., threshold exceedances).

As geo-referenced data is subject for further processing, one important aspect is to control its data quality. Quality criteria comprise lineage, logical consistency, completeness, temporal quality, or spatial accuracy. From a practical viewpoint, this means that the location may be used to define quality indicators which can be integrated into CEP pattern rules. This happens by the use of external geographical code libraries, as presented in Subsection 5.3.

Within the Live Geography approach, it is primarily used for performing operations on events and synchronously derived event data (e.g., sensor fusion results). These operations comprise—amongst others—quality assurance like filtering and error detection, whereas more complex processing is performed by data fusion and geo-processing operations. In effect, this means that the CEP component can trigger a geo-processing or data-fusion operation based on an event that appears in fused data.

### 4.4. Integration of SAS and CEP

As described in Subsection 4.2.4, the OGC Sensor Alert Service (SAS) specifies interfaces (not a service in the traditional sense) enabling sensors to advertise and publish alerts including according metadata. In the SAS specification, “alerts” are not only understood in the classic meaning of the word, *i.e.*, an automatic signal or notification indicating that an event has fired (e.g., a message in case of threshold exceedance), but in a broader context. Alerts are defined as “data” sent from the SAS to the client, which may as well comprise alerts/notifications (e.g., OGC WNS) as observational data (measurements matching pre-defined criteria).

From an implementation viewpoint, SAS may be integrated with a CEP system as it operates asynchronously—after a synchronous initialisation process. An adapter for the service manages the subscriptions and converts received alerts into events that can be processed by the filtering engine. The adapter itself maintains a list of all established connections. A connection comprises several subscriptions and their corresponding chat rooms (MUC). In order to retrieve a sensor subscription, the adapter sends a request. This request can be used for all three types of service operations—*Subscribe*, *RenewSubscription* and *Unsubscribe*. After the CEP process has finished, the request object fires a service event containing the result of the operation. If the operation was successful, the SAS adapter adds the subscription data to its list and joins the returned chat room.

A challenging particularity is that SAS lacks advanced filtering capabilities. Thus, CEP rules cannot be matched directly onto the SAS interface. This issue is resolved in that state-of-the-art CEP engines rely on adapters to fetch the data that should be processed. Such an adapter has been used to create a pre-selection layer transforming data into pre-defined event types - as only pre-processed information can be parsed by a CEP system. Basically the adapter is connected to the SAS and uses the small set of provided filtering capabilities to create a further subset of data for CEP. Since a central CEP issue is handling huge amounts of data in relatively short time, this pre-selection can be used to narrow down the relevant dataset. The wide range of CEP capabilities can then be applied to this reduced set. Moreover, a CEP module is needed to connect the CEP engine with the data adapter and the resulting complex events. Within this research, a prototype has been implemented using the Esper CEP engine. This technology choice is motivated in Subsection 5.3.

For the integration of geographic topological functions in the filtering process, it is necessary to offer a possibility to define these functions when entering a CEP rule. The Esper CEP engine provides this functionality in the form of User-Defined Functions (UDF). A set of topological rules (based on open-source Java Topology Suite) has been implemented for OGC Simple Features, as described in Subsection 5.3.

Concluding, it shall be stated that SAS can be combined with CEP in a very modular fashion allowing for loose coupling of event processing and alerting/notification components. A future point of discussion is the integration of CEP and alerting with the OGC Web Processing Service (WPS). This issue is however not in the scope of this paper.

## 5. Intelligent Sensing Device Implementation

The implementation described within this section comprises a tailor-made sensing device, embedded implementations of SOS and SAS services, a CEP and alerting module, and an optimised database schema. All these components are unified on an embedded device (as explained below) and make up an intelligent sensor pod. The underlying scientific challenge is the conception of a framework, which allows for the integration of standardised pre-processed sensor measurements into GIS (Geographic Information Systems).

### 5.1. Embedded Sensing Device

The measurement device has been particularly designed for pervasive monitoring applications using ubiquitous embedded sensing technologies. The system has been conceived in such a modular way that the base platform can be used within a variety of sensor web applications such as environmental monitoring, biometric parameter surveillance, critical infrastructure protection, or energy network observation by simply changing the interfaced sensors.

**Figure 5.**
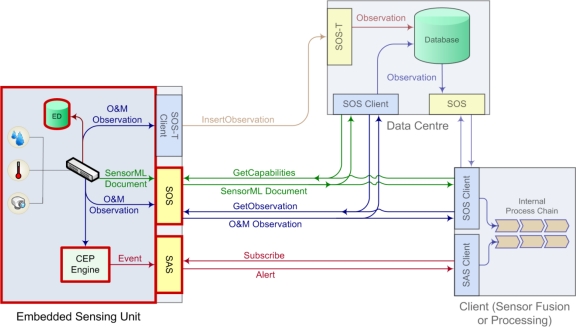
Communication of the Sensor Device with other Components in the Workflow.

As it can be seen from Figure 5, the communication of the sensing device with other components in the workflow occurs on the basis of open standards of the Sensor Web Enablement (SWE) family. This requires a SensorML-conformal description of the measurement platform, O&M-compliant encapsulation of measurement values, as well as an SAS-compliant alerting module, which is separately explained in Subsection 5.2.2. In addition, a CEP engine implementing event-based data processing has been integrated directly on the embedded device.

Furthermore, a lightweight database is utilised on the sensor device to provide for the possibility of short-term data storage. This enables trend analysis and quality assurance, and reduces communication overhead with the central archive database.

The intelligent sensor pod implements the most essential standards of the SWE family, as illustrated in Figure 5. These standards have been described in more detail in Subsection 4.2. The detailed software and service infrastructure running on the embedded sensing device is described in Subsection 5.1.2.

The pod connects sensors via a variety of hardware interfaces such as serial, UART (Universal Asynchronous Receiver/Transmitter), I2C (Inter-Integrated Circuit), Universal Serial Bus (USB), ZigBee, Bluetooth *etc*., stores a short history of measurement data in the embedded database and serves data over the internet using a secure web server (HTTP, HTTPS) or a chat service (XMPP). This standardised data provision can either happen using SOS (providing measurement data in O&M format) or SAS (providing alert data in an asynchronous fashion).

#### 5.1.1. Sensor Pod Hardware

Compared to general-purpose computers (e.g., PCs), embedded devices are particularly characterised by their specific functionality and their real-time capabilities. Thus, embedded devices are employed in a wide variety of applications, ranging from MP3 players to industrial control devices, building automation, or automated diagnosis systems in the automotive sector. The main characteristics of embedded devices are thus:

- tailored design for a specific field of application
- integrative design
- minimised costs
- low energy consumption
- optimised software infrastructure
- constrained capabilities of the hardware infrastructure

**Figure 6.**
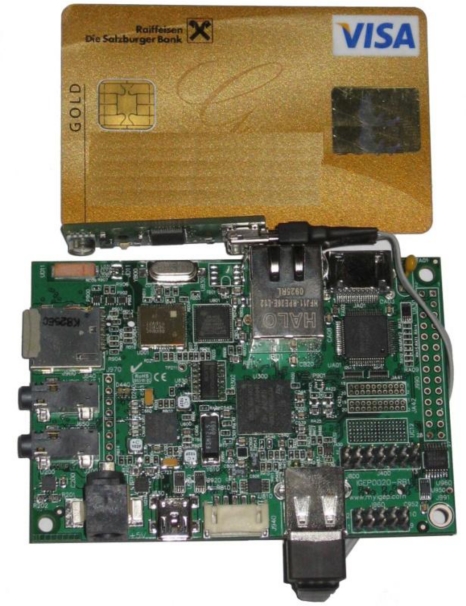
ISEE IGEPv2 with GPS and a Combined Air Temperature and Humidity Sensor.

The sensor pod used in this implementation consists of a COTS embedded device, an ISEE IGEPv2 platform including an ARM7-based Cortex A8 600 MHz processor with 512 MB RAM and 32 MB flash memory, as shown in Figure 6. Generally speaking, ISEE offers a highly modular and easily expandable system. The computer-on-module (the actual embedded device including CPU, memory and several interfaces) offers two I/O ports, which allows for extensibility of the basic system by specific modules such as GPS, Bluetooth, Wireless Fidelity (WiFi), Local Area Network (LAN), interface breakouts or a console board for programming the device.

In the configuration for this specific implementation, a GPS module (U-BLOX NEO 4S and LEA-4P) has been attached for positioning. Different sensors (e.g., LM92 temperature, Toradex Oak USB for air temperature and humidity, NONIN 8000SM oxygen saturation and pulse, or SSM1 radiation sensors) have been interfaced via standardised interfaces like UART, I2C, USB *etc*. The configuration of the board happens via the Serial Port Debug interface. This enables the establishment of a connection to the embedded device via the RS232 port for installing the bootloader, the operating system and other software, as well as for debugging. The following figure shows the ready-to-deploy embedded device including the main computation component, a Toradex Oak USB air temperature and humidity sensor and a GPS module. A customary Universal Mobile Telecommunications System (UMTS) dongle for communication is attached to a further micro-USB port.

The size of the complete sensor pod is approximately 93 × 65 × 10 mm, *i.e.*, about the size of a credit card. As the used device is a COTS product, it can still be optimised in size, interfaces and hardware specifications according to specific application requirements. In full load, the device features an energy consumption of <2.2 W including a running data query, the GPS module and data transmission via UMTS, which is known to be comparatively energy intensive way of broadcasting data. The following calculation yields an operation time under full load of 9.1 hours given a battery capacity of 4,000 mAh:

(1) $$\begin{array}{l} Q\ldots 4000mAh\\ P\ldots 2.2W\\ U\ldots 5V\\ P=U\cdot I\Leftrightarrow I=\frac{P}{U}\\ Q=I\cdot t\Leftrightarrow t=\frac{Q}{I}=\frac{Q\cdot U}{P}\\ \Rightarrow t=\frac{4000mAh\cdot 5V}{2.2W}=9.1h \end{array}$$

The electric charge is defined as the product of current conduction and time (*Q* = *I·t*), which in turn means that the time “a battery lasts” is the electric charge divided by the current conduction ( $t=\frac{Q}{I}$). As the current flow can be expressed as electric power over voltage ( $I=\frac{P}{U}$), *i.e.*, *1 W = 1 VA*, time can be calculated by the term $t=\frac{Q\cdot U}{P}$.

The above calculation was done using an assumed electric charge of 4,000 mAh, which is held by a reasonably-sized rechargeable Lithium-ion Polymer (LiPo) battery (140 × 40 × 10 mm)—where its capacity and size is naturally dependent on the specific use case—and a constant voltage of 5 V as per the specifications of the embedded device. In a production environment, the sensor pod shuts down non-active components (transmission unit, GPS receiver *etc*.) in order to increase battery lifetime.

#### 5.1.2. Software Infrastructure

**Figure 7.**
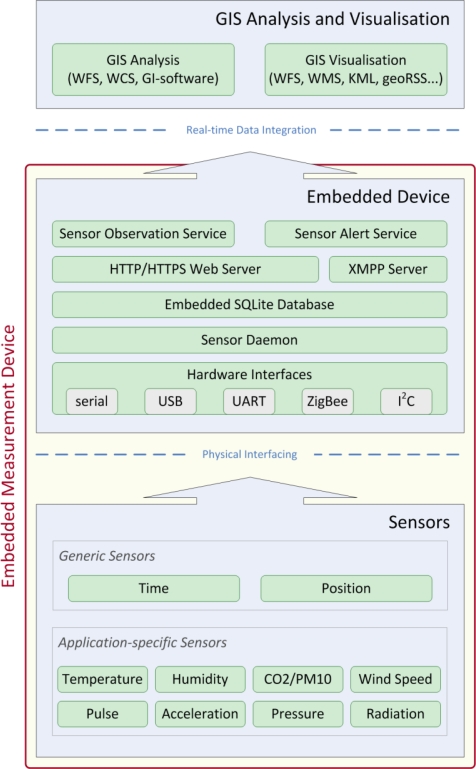
Software Infrastructure on the Embedded Sensing Device.

The sensing device runs a customised version of the *Ångström* Linux distribution that is generated by the cross-compile tool-chain “Open Embedded”. The used kernel version is 2.6.33 with an approximate footprint of 2 MB. The basic operation system’s footprint is about 10 MB. This operating system houses a software infrastructure, which contains several services, daemons and hardware drivers, as shown in Figure 7. The software infrastructure comprises an embedded secure web server (Lighttpd), an SQLite database and several daemons, which convert sensor readings before they are served to the web. The database acts as short-term storage of historic measurements to allow for error detection procedures and plausibility checks, as well as for non-sophisticated trend analysis.

The hardware drivers for interfacing sensors and reading their measurements make up the first part of the embedded software infrastructure. As the geographical position is an essential must-parameter in geo-sensor webs, the sensor pod always interfaces a location sensor (e.g., a GPS/Galileo module, a ZigBee/WiFi-based positioning component *etc*.) or uses a cached position if no other option is available.

These measurements are then read by a special sensor daemon that essentially builds the bridge between the sensors and the internal software components. The daemon, which is running in the background, interprets the bit stream coming from the sensor interface and converts it into meaningful measurement data.

These data are then stored into an embedded database (*SQLite*), which is held at a maximum data set volume, currently 12.500 readings. The database implementation and the relational data model will be described separately in Subsection 5.1.3. The sensor data, which are stored in the database, are then provided by either a web server (HTTP/HTTPS) or an XMPP-based MUC server, which make the measurements accessible from the internet.

HTTPS is considered a high enough security level for this implementation providing a secure channel between server and client using the Secure Socket Layer (SSL) protocol. Web Service Security (WSS), would be a viable alternative providing message-based security. However, as WSS is using the SOAP, it is characterised by large overhead, which is not well suitable for embedded sensor unit implementations.

The *Lighttpd* web server was chosen as HTTP/HTTPS server because of its very compact design and its low resource requirements. Its minimal footprint of a few kilobytes flash memory usage highly qualifies the server for use with embedded devices. Furthermore, Common Gateway Interface (CGI) functionality is integrated, which is essential for providing sensor data via the internet. Lighttpd uses a 256 Bit Advanced Encryption Standard (AES) encryption for securing HTTP connections to a client.

Due to its small footprint and open-source availability, the *jabberd2* Extensible Messaging and Presence Protocol (XMPP) server was chosen for the implementation of alerting functionality on the embedded device. XMPP is an Internet Engineering Task Force (IETF) standard for push-based communications technology. It was designed for real-time XML-based communication, enabling a broad spectrum of applications such as instant messaging, media negotiation, collaboration and more.

As alerting functionality including CEP and SAS implementations is a vital part of the workflow, it is treated separately in 6.3.

Finally, the “top” of the software stack in Figure 7 consists of two service interfaces towards the internet. The OGC SOS and SAS make sensor data available over the web. The actual embedded implementations of SOS and SAS will be described in Subsection 5.2.

#### 5.1.3. Embedded Database

In order to allow for short-term storage of sensor measurements, the sensor pod runs an embedded database. It basically serves for organisation of a small amount of data sets (e.g., 12.500 entries as explained below) to enable quality control, error detection or trend analysis, directly on the embedded device.

The requirements for the implementation of an embedded database are a minimal footprint and support for the quasi-standardised Structured Query Language (SQL). Thus, SQLite was chosen, which is a “self-contained, server-less, zero-configuration, transactional SQL database engine” [16]. SQLite is an in-process database, meaning that it does not have a separate server process. Furthermore, the database file format is cross-platform qualified enabling deployment on various operating systems and hardware platforms.

**Figure 8.**
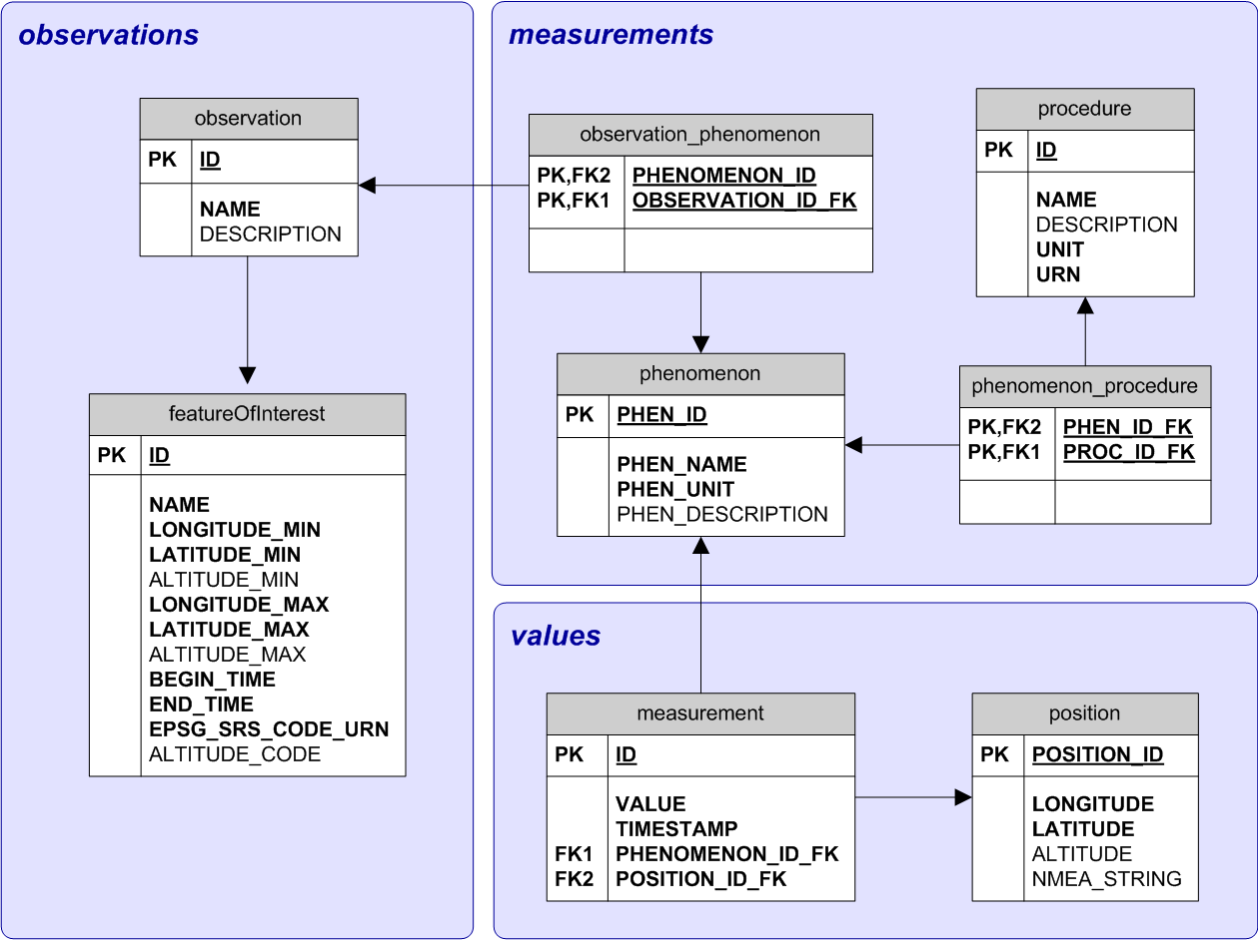
Relational Schema of the Embedded Measurement Database Structure.

The initial realisation of an OGC-conformant Sensor Observation Service (SOS) is done by the open-source initiative 52° North [17]. As this database model, which is currently a reference implementation, is using 22 tables, a lot of constraints and a high degree of normalisation, it is too complex to achieve reasonable performance, particularly for implementation in embedded databases. Thus, it was decided to create a separate schema for the Live Geography implementation, as illustrated in Figure 8.

The model is divided into three categories of sensor data sub-sets. Firstly, the observation part contains the observation itself and the feature of interest; secondly, the measurements section comprises the phenomena and procedures; and thirdly the values set covers the measurement values itself including their position. These terms are defined in Subsection 5.2.1.

The embedded database schema has been designed towards simplifying SOS queries while aiming for an optimal trade-off between normalisation, completeness and performance. The element structure is highly similar to SOS process logic and to O&M data structures. For the Live Geography implementation, the database holds 12.500 measurement values, which approximately corresponds to 1 MB of data volume presuming that one dataset is 80 bytes. This limit was set to account for the specific sensor device’s limited storage capacities and can naturally be adapted for other use cases. The presented relational schema is reasonably scalable as it uses few constraints, but still corresponds to the 4th normal form. In order to prevent m:n-relations, interim tables have been inserted to ensure data integrity.

To allow for longer-term data analysis, the measurements stored directly on the sensing device have to be transferred to an archive database, which is housed in a central data warehouse. However, this collection database has not been set-up within the implementation phase of this research as this is a standard process without major research aspect. Several spatial relational database products offer ready-to-use automated synchronisation mechanisms. Thus, it was not necessary to develop a dedicated software component for this purpose. However, it shall be noted that a database storing historical measurement data is naturally essential for sophisticated longer-term analysis in the context of fully-fledged Spatial Data Infrastructures (SDI).

### 5.2. Standardised Service Infrastructure

For standardised data retrieval, a service has been created implementing the OGC Sensor Observation Service (SOS), Sensor Model Language (SensorML) and Observations and Measurements (O&M) standards in an application-specific way. Like this, measurement data are served via HTTP/HTTPS over UMTS in the standardised XML-based O&M format over the SOS service interface. SensorML is used to describe the whole sensor platform as well as to control the sensor via the OGC Sensor Planning Service (SPS), e.g., to dynamically adjust measurement cycles. It shall be mentioned that SPS has to date only been implemented in a rudimentary prototype. Finally, SAS is responsible for creating events and alerts from measurement data. The overall service stack on the embedded sensing device is illustrated in Figure 7.

#### 5.2.1. Embedded OGC Sensor Observation Service

The OGC Sensor Observation Service (SOS) serves for querying measurement data from the embedded sensing unit. The service implements the three mandatory methods as specified by the OGC SOS standard [12], *DescribeSensor*, *GetCapabilites* and *GetObservation*. Basically, the service, which is implemented in CGI, parses the request and creates the according response using appropriate XML templates.

**Figure 9.**
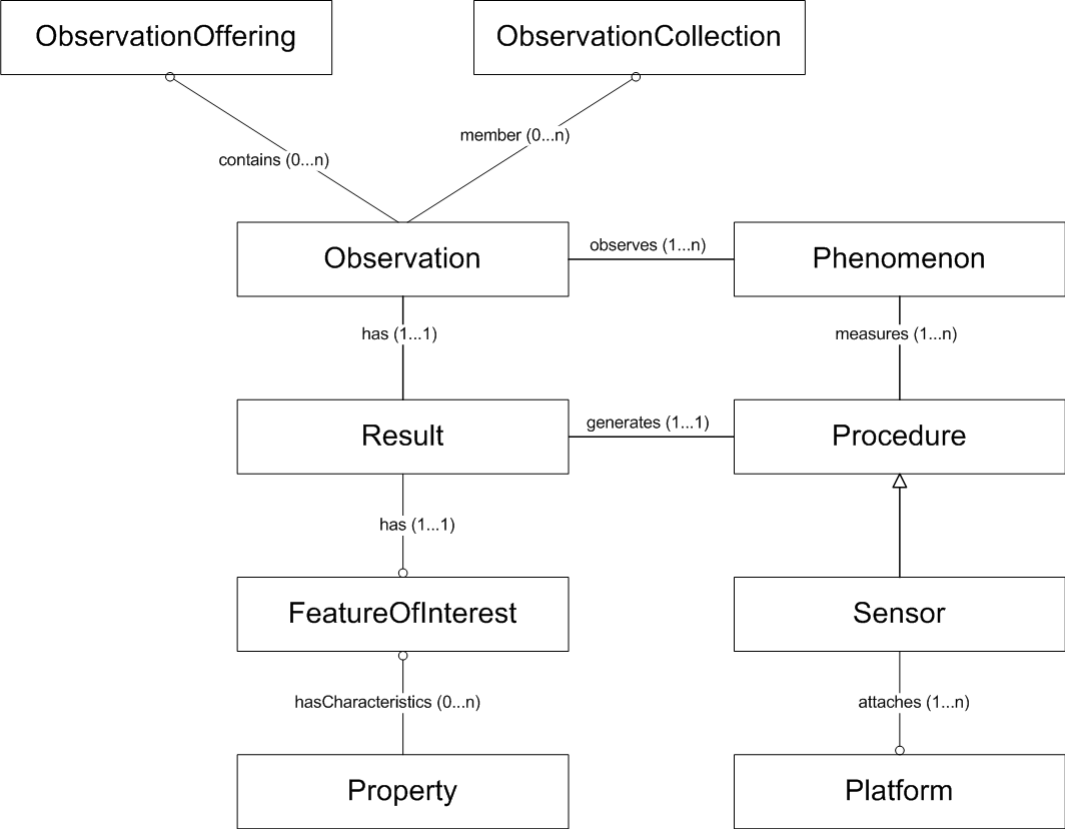
OGC Data Model for O&M.

The *DescribeSensor* operation returns a SensorML-conformant description of the sensor platform. The *GetCapabilities* method provides a SOS capabilities document including identification, input and output lists, contact data and measurement metadata, amongst others. The *GetObservation* function queries the embedded database according to the *GetObservation* request and returns the required measurement data in the standardised O&M format. The O&M data structure is shown in Figure 9. This structure is a custom Live Geography development for embedded applications and is optimised for the implementation presented in this paper. It shall be mentioned that this is a simplified view on the connection of O&M elements to clarify their basic interplay, without claiming completeness as specified by the OGC O&M standard.

According to the OGC O&M standard [11], the elements in Figure 9 are characterised as follows:

- observation—an act of observing a property or phenomenon, with the goal of producing an estimate of the value of the property. A specialised event whose result is a data value.
- phenomenon—concept that is a characteristic of one or more feature types, the value for which may be estimated by application of some procedure in an observation.
- property—characteristic of a feature type, including attribute, association role, defined behaviour, feature association, specialisation, and generalisation relationship and constraints.
- procedure—method, algorithm or instrument. It is the description of a process used to generate the result. It must be suitable for the observed property.
- result—an estimate of the value of some property generated by a known procedure.
- featureOfInterest—a feature (abstraction of a real-world phenomenon) of any type, which is a representation of the observation target, being the real-world object, regarding which the observation is made.

#### 5.2.2. Embedded OGC Sensor Alert Service

The implemented Sensor Alert Service (SAS) is responsible for sending alerts, which have been triggered by events created in the CEP engine, as explained in Subsection 5.3. The service implements the mandatory operations according to the specification, namely *DescribeSensor*, *DescribeAlert*, *GetCapabilities*, *Subscribe*, *RenewSubscription* and *CancelSubscription*, as illustrated in [13].

The *DescribeSensor* and *GetCapabilities* operations are used analogically to the SOS methods described in Subsection 5.2.1. The *DescribeAlert* method serves for requesting a template of the alert message structure.

The *Subscribe* operation allows users to subscribe to a particular sensor alert, *i.e.*, to receive a notification if a pre-defined rule applies. If a user registers for an alert, the sensor returns a *SubscribeResponse* indicating the XMPP channels used for messages and acknowledgments. Moreover, the response includes an expiration timestamp, until the data may be retrieved, and a unique ID for identifying the subscription.

As its name indicates, the *RenewSubscription* operation allows clients to renew their subscription to a sensor alert before it expires. In case such a request is issued, the sensor automatically extends the expiration time and responds with a *RenewSubscriptionResponse*. Accordingly, a client can also cancel a subscription by sending a *CancelSubscription* request. A more detailed description of the SAS implementation can be found in Subsection 5.3, as this module is closely coupled with the CEP component.

### 5.3. CEP and Location-Aware Complex Event Processing and Alerting

Furthermore, a CEP and alerting mechanism based on Extensible Messaging and Presence Protocol (XMPP), conformant to the OGC Sensor Alerting Service (SAS) specification has been implemented. For generating alerts, the SAS standard has been implemented for mobile sensor devices. SAS, which is part of the SWE initiative, specifies interfaces allowing sensors to advertise and publish alerts including according metadata.

Alerts, which are based on SAS filter rules, are defined as “data” sent from the SAS to the client or, in this case, to a Complex Event Processing Engine (CEP). Data can either be alerts/notifications (e.g., OGC WNS) or actual sensing data (measurements matching pre-defined criteria). As SAS is based on the standardised XMPP protocol, alerts can be broadcasted very efficiently over the internet to subscribed consumers [13]. A typical SAS-compliant alert is shown in the following XML structure (2). The alert data can be parsed according to the structure defined in the *DescribeAlert* response.
(2)
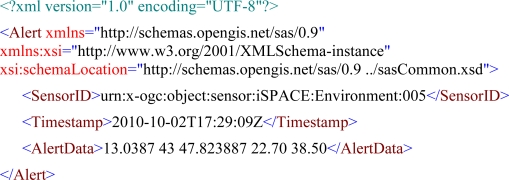


Within the Live Geography concept, CEP is used for detecting patterns in measurement data and for creating complex events accordingly. These can be related to time, space or measurement parameters, as explained in Subsection 4.3. These CEP operations can occur at two different stages of the workflow. Firstly, at sensor level CEP is used to detect errors in measurement values by performing different statistical calculations including standard deviations, spatial and temporal averaging, or outlier detection. Secondly, after the data harmonisation process CEP serves for spatio-temporal pattern recognition, anomaly detection, and alert generation in case of threshold transgression.

For sending events created by the CEP engine, a push-based SAS compliant alerting service has been realised. In this case, SAS is an asynchronous service connecting a sensor in a network to an observation client. If specified rules apply, a pre-defined alert is sent to subscribed clients via XMPP. It shall be stated that the whole communication between the embedded XMPP server (jabberd2) and the client is XML-based for simplifying machine-to-machine (M2M) messaging.

**Figure 10.**
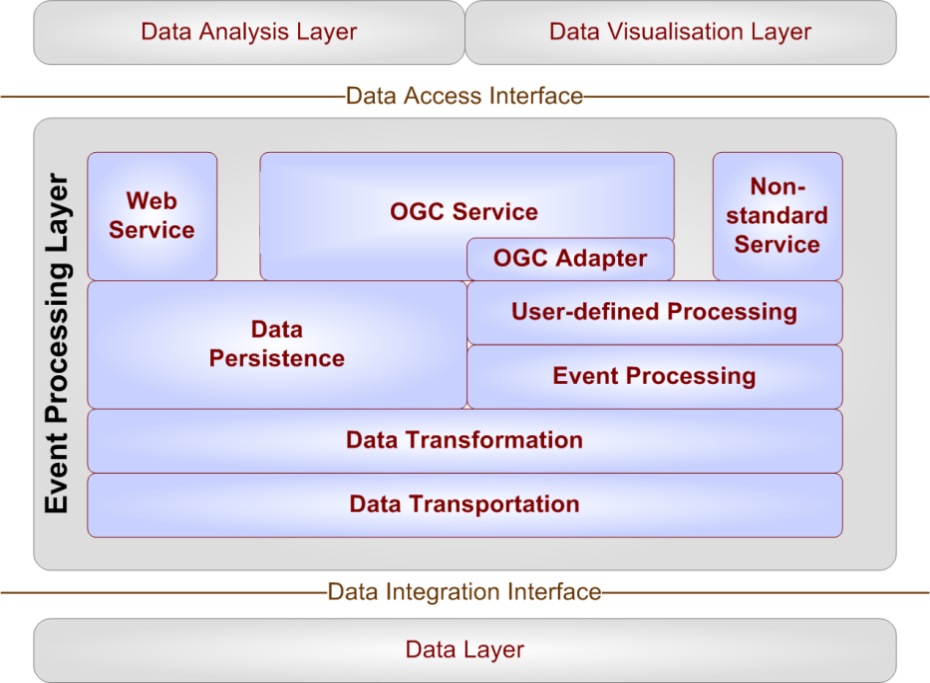
Internal Service Stack of the CEP Component.

In the actual implementation, the Esper CEP engine in its version 3.0.0 has been used because of its open availability, Event Query Language (EQL) based event description, and its simple integration by just including a single Java library. The evaluation of available geographic code libraries yielded that the Java Topology Suite (JTS) is the preferable solution for this implementation because of its easy integration. This choice has been taken despite GeoTools offers a richer feature set. However, JTS’ features are more than enough for location-aware CEP implementations. Figure 10 shows the internal sub-parts of the CEP-based event processing component, which is built up in a modular structure.

Generally speaking, the event processing component connects the data layer (*i.e.*, sensor measurements), with data analysis and data visualisation components. In other words, it prepares raw data to be process-able in the analysis and the visualisation layers.

The **data transportation** component connects the data integration layer to a variety of data sources, which comprise sensor data, real-time Really Simple Syndication (RSS) feeds, File Transfer Protocol (FTP) services, web services, databases *etc*. Thus, it can be seen as an entry point into the system. Its main task is to receive different kinds of data structures and pass them on as is to the data transportation layer adding metadata do the actual payload, such as the data source or its format.

The **data transformation** component receives the data stream from the transportation module and parses it. This component transforms the data into objects in a pre-defined way. After this conversion procedure to a commonly understandable data format or a class instance, the component passes the object on to the event processing engine or the data persistence component depending on pre-defined criteria such as the type of object, its origin or the data format.

The **event processing** component deals with the objects coming from the transformation module according to specified user query statements. It basically manages multiple streams, and identifies and selects meaningful events. In the implementation presented in this paper, the Esper event processing engine is used to detect events and push data to the user processing component. The Esper engine has been extended by spatial parameters as described above. Following the event processing step, further user-defined processing can optionally be applied. This happens through a set of filtering or selection rules, which are specified by the user according to a very particular need.

The **data persistence** component receives a set of data from the data transformation module or from the processing modules (*i.e.*, a “filtered” dataset). From these data, it established a physical data structure, which can either be temporary (for rapidly changing real-time data) or permanent (for time-insensitive data, e.g., for stationary sensor locations). Consequently, as well live data sources as static ones can be handled by the data persistence component.

The **non-standard service** is one of three service interfaces, which connect the event processing component to the data analysis layer. It provides data via a custom (*i.e.*, non-standard) interface. This is necessary as existing standardised OGC services do not automatically support push mechanisms. Naturally, standardised data structures can also be served via the non-standard interface. The output can either be e.g., GML, Keyhole Markup Language (KML) or GeoRSS for spatial data, or RSS, JSON, SOAP bindings or a custom API for non-spatial data.

The **OGC service** is the second component, which connects the data CEP layer to the data analysis layer. It offers well-known and widely spread standardised interfaces in proved data structures such as GML, GeoTIFF, KML or SVG. The indicated **OGC real-time adapter** is basically a technological bridge to integrate live data into existing OGC services as existing implementations only support a variety of quasi-static data sources such as shape files, ASCII grids, different (geospatial) databases or cascading OGC services (WFS, WMS, WCS). Its implementation has been described in [3].

Finally, the **web service** component is intended for handling non-geographic non-real-time data and for serving it via the http protocol. This component is basically a regular web service, meaning that it implements the request/response communication model, but no push-based mechanism.

### 5.4. Performance Evaluation

The SOS service is the main interface for data provision. It follows the classical OGC Web Services (OWS) pull-based request-response model as defined by OGC—see Subsection 5.1.2 for more detailed information. For near real-time applications, the service’s performance is essential. Thus, the sensing device has been tested under the following pre-conditions:

- The database on the embedded device contained 1,000 measurement data sets.
- All datasets were queried.
- The *GetObservation* data request was sent via HTTP POST.
- IP-based communication happened over UMTS.
- The pod’s IP address was mapped via DynDNS (http://www.dyndns.com).
- Position information was captured via DPGS enhanced by NTRIP correction parameters.
- Qualitative measurements were collected using the radiation sensor SSM-1, which was connected to the embedded device via the RS232 interface.
- During the whole test, measurements (time, position and radiation dose rate) were recorded at a one-second interval.
- A number of 100 data queries were performed over an interval of five minutes.

The tests yielded an average response time of 1.9 seconds, where the size of the O&M-encoded SOS response was approximately 38 kB. The measured response time includes sending the request on the client side, parsing it on the server side, querying the embedded database, encapsulating the measurements in O&M-conformal format, and sending the *GetObservation* response, including error handling. At the same time, measurements were recorded by the sensor pod every second, querying the sensor over the serial interface, reading the DGPS position including NTRIP correction, and updating the database, which was kept at the constant size of 1.000 datasets as mentioned above.

These performance figures show that the presented intelligent sensor pod is capable of providing measurement data in near real-time. It shall be mentioned that the IP address mapping through DynDNS and data transmission via the cellular network introduce latencies, which could be mitigated by the use of alternative communication technologies such as WLAN. However, the goal of these tests was to evaluate the system’s performance in a realistic real-world scenario instead of in an optimised lab environment.

## 6. Assessment of Standardisation Initiatives and Impacts on Pervasive Monitoring

As mentioned in Subsection 3.1, standardisation is a vital prerequisite for creating interoperable and portable infrastructures. To achieve maximum interoperability, standardisation has to be applied throughout the whole workflow, ranging from unified data encapsulation and representation to service interfaces for data provision, event detection and alert generation, and to workflow management.

### 6.1. Standardisation Initiatives

**Figure 11.**
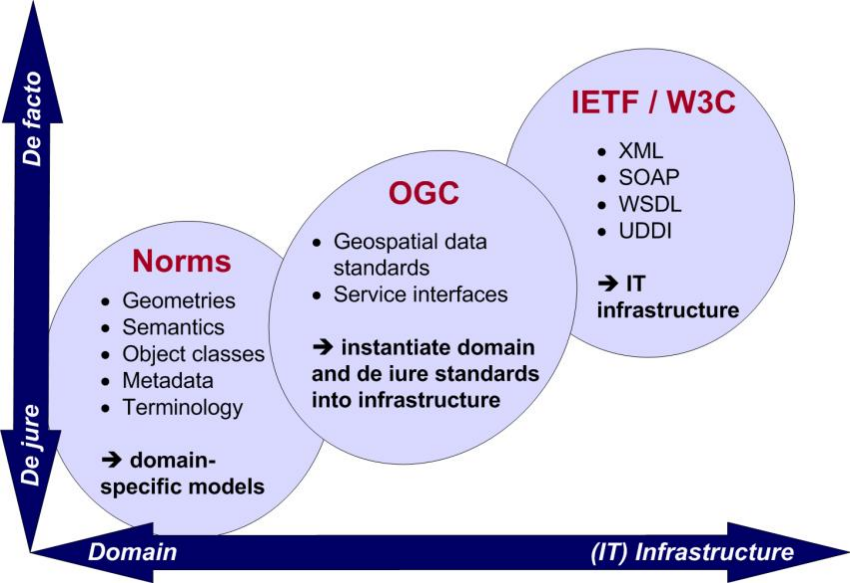
Standardisation Bodies Classified by Legal Bindingness and Genericness.

As shown in Figure 11, there are a variety of standardisation efforts by different institutions aiming at interoperability of (geospatial) data and services. Basic infrastructure-related standards (e.g., World Wide Web Consortium (W3C) web service or communication standards) are mostly “de facto” standards (commonly used technological specifications). On the contrary, domain-specific definitions are mostly legally binding (“de jure”). As de facto standards are often developed “on-demand”, *i.e.*, by a specific group of mostly industrial companies, and norms are often imposed by legislation, the OGC tries to bridge these two worlds. This happens by creating service interfaces using established de facto standards while still being open for incorporating legally binding domain-specific norms.

The OGC pursues a double-sided approach in establishing geospatial standards. General standards are developed in a top-down fashion. For instance, the GML standard has been created aiming to model every single object in the world while being as generic as possible. In contrast, standards such as KML have been created out of a concrete need of specialised companies (in the case of KML, Google needed a format for data representation for their 3D viewer Google Earth). In case of broad support and development perspectives, these formats are then officially adopted as OGC standards.

### 6.2. Benefits Arising from the Usage of Standards

The establishment of open architectures can benefit in various ways from the use of open standards. First, standardised data representation/provision, and information visualisation foster **interoperability** between services and heterogeneous data sources. Like this, different end users can be served by providing intuitive and tailored graphical interfaces by decoupling data and visualisation. This interoperability also allows for automated **M2M** communication via well-known interfaces. Thus, various services can be combined to achieve modular service-chaining. This results in wide reusability of different software components, which can be easily adapted to perform different tasks.

Another crucial benefit of using open standards is simple **extensibility** in terms of integrating new data sources and analysis components into a modular system. This means that business processes benefit by this straight-forward integrability of additional resources. Furthermore, uniform interfaces ensure **request consistency**, facilitating the implementation of client applications. Subsequently, well-established geo-interfaces like OGC WFS, WMS or WCS ensure preservation of request structures in contrast to custom APIs, which may change frequently due to newly arising application needs.

One trade-off when using open standards is that **increased effort** is needed to establish the system when first deploying it. The time-consuming part is to implement standards, which can be widely avoided in the GIS domain by using open-source software where applicable. However, on the long-term, operation, administration and transaction **costs are minimised** using open standards as maintenance and communication are much simpler and cheaper, as stated in [18] and [19].

Summarising, it can be stated that open standards in pervasive measurement infrastructures foster interoperability and reusability. The main goal is to achieve request uniformity within the system. Moreover, the integration of new and emerging standards such as OGC SWE is a main focus in the sensor web part of the Live Geography approach. This allows for automated M2M communication as sensor data can be automatically integrated into real-time analysis processes.

### 6.3. Techno-Political Issues—Standardisation Enables Open Measurement Infrastructures

The widespread availability of sensor data with high spatial and temporal resolution is expected to increase dramatically with rapidly decreasing sensor prices [3]. One way to address this issue is the extensive use of open geospatial standards for structuring and managing heterogeneous data. The key technological challenge is the integration of vast amounts of real-time data sources owned by public bodies, governmental institutions and private sensor network operators. This problem can be tackled with self-contained data encapsulation standards—independent of specific applications—and enforced by legal entities. However, the adaptation of existing sensors to new standards is costly for network operators in the short term. Hence, increased awareness of the benefits of open standards is required.

From a technical viewpoint, unresolved research challenges for ubiquitous monitoring infrastructures are manifold and include: finding a uniform representation method for measurement values, optimising data routing algorithms in multi-hop networks, data fusion, and developing optimal data visualisation and presentation methods. The latter issue is an essential aspect of decision support systems, as different user groups might need different views of the underlying information. Naturally, there are a number of well-known domain-specific technical issues (energy supply, mote size, robustness, routing, ad-hoc network connections, connectivity, self-healing mechanisms *etc*.). These have to be addressed depending on specific end application requirements. Moreover, highly unpredictable challenges exist arising from openly accessible, dynamic and variable environments, such as severe weather conditions, malfunctioning hardware, connectivity, or even theft and vandalism.

From a political and legal standpoint, national and international legislative bodies are called upon to foster the introduction of open standards in public institutions. Comprehensive efforts in this direction are made by the European Commission through targeted directives such as INSPIRE, which aims at Europe-wide harmonisation of discovery and usage of geo-data for analysing and solving environmental issues [20]. These regulations support the establishment of ubiquitous and generically applicable real-time data integration mechanisms. Shifting development away from proprietary single-purpose systems towards interoperable analysis infrastructures may enable live assessment of our surroundings and can lead to a new perception of our environment. Consequently, this trend may foster the creation of innovative applications for interactive sensing and participation platforms. One example is the WikiCity concept [21], involving people themselves into re-shaping the urban context.

## 7. Conclusions and Future Outlook

This paper presents the technical infrastructure for an intelligent standardised embedded sensing device, which is part of the Live Geography approach. The goal of this research is that the resultant highly flexible measurement and analysis architecture will bring sensor network applications one step further towards the realisation of the vision of a “digital skin for planet earth”. The broad objective of this research is to develop an overarching infrastructure for various kinds of sensor web applications. The developed architecture can potentially have far-reaching impacts on sensor-based monitoring systems through the deployment of ubiquitous and fine-grained sensor webs.

A vital prerequisite to achieve this comprehensive vision is extensive use of open standards in measurement data provision, sensor fusion, analysis and visualisation. Unlike proprietary monolithic systems, this enables automated M2M communication across multiple application domains. Thus, such an approach supports the occurring paradigm shift in GIS that the creation of added value is not revolving around data anymore, but around processes, and increasingly around (information) services.

The intelligent sensor pod presented in this paper uses a wide range of (geospatial) standards such as OGC’s SOS, SAS in connection with Complex Event Processing (CEP), SensorML, O&M, GML, and KML. This addresses current compatibility issues between existing and new sensor networks by creating of a common communication framework. The long-term goal is to support the establishment of pervasive sensor webs, providing the basis for ubiquitous and holistic information services.

The particular novelties of our approach can be summarised as follows:

- *Mobile SWE*: as opposed to existing approaches, the OGC SWE suite has been implemented on a COTS embedded device to achieve a significant leap towards the vision of pervasive sensing.
- *Technology Bridge*: the Live Geography approach builds an architectural bridge between domain-independent technical sensor network development and use case specific requirements for end user tailored information output.
- *Interoperable Geo-sensing*: providing standardised sensor service interfaces allows for the establishment of ubiquitous information services by making heterogeneous data sources combinable through well-defined request parameters and data structures.
- *Location-aware CEP*: existing CEP concepts have been extended by spatial parameters enabling the implementation of quasi-standardised pattern matching in geospatial data, e.g., for detecting threshold exceedances, for geo-fencing implementations, for investigating spatial clusters or as well for assuring data quality.
- *Standardised Alerting*: coupling the OGS SAS service interface with location-aware CEP mechanisms allows for generating automated alerts (according to pre-defined criteria) and their transmission via a variety of notification channels.

Two main challenges in geo-sensor web research in the coming years will be to harmonise existing networks with upcoming efforts, and to establish automated sensor discovery mechanisms as proposed in [22]. This guarantees optimal data availability for assessing environmental dynamics. However, achieving this vision presumes a shift from developing monolithic single-purpose sensor systems towards creating interoperable measurement infrastructures, which requires adequate public awareness and policy frameworks. This in turn allows for the straight-forward use of live sensor data in existing spatial decision support systems to enable better-informed decision-making.
